# A Reliable Prediction Model for Renal Cell Carcinoma Subtype Based on Radiomic Features from 3D Multiphase Enhanced CT Images

**DOI:** 10.1155/2021/6595212

**Published:** 2021-09-21

**Authors:** Haijie Zhang, Fu Yin, Menglin Chen, Anqi Qi, Zihao Lai, Liyang Yang, Ge Wen

**Affiliations:** ^1^Medical Imaging Department, Nanfang Hospital, Southern Medical University, No. 1023, South Shatai Road, Baiyun District, Guangzhou 510515, China; ^2^PET/CT Center, The First Affiliated Hospital of Shenzhen University, Shenzhen Second People's Hospital, No. 3002, West Sungang Road, Futian District, Shenzhen 518052, China; ^3^College of Information Engineering, Shenzhen University, No. 3688, Nanhai Road, Nanshan District, Shenzhen 518052, China

## Abstract

**Background:**

This study aimed to develop a prediction model to distinguish renal cell carcinoma (RCC) subtypes.

**Methods:**

The radiomic features (RFs) from 5 different computed tomography (CT) phases were used in the prediction models: noncontrast phase (NCP), corticomedullary phase (CMP), nephrographic phase (NP), excretory phase (EP), and all-phase (ALL-P).

**Results:**

For the ALL-P model, all of the RFs obtained from the 4 single-phase images were combined to 420 RFs. The ALL-P model performed the best of all models, with an accuracy of 0.80; the sensitivity and specificity for clear cell RCC (ccRCC) were 0.85 and 0.83; those for papillary RCC (pRCC) were 0.60 and 0.91; those for chromophobe RCC (cRCC) were 0.66 and 0.91, respectively. Binary classification experiments showed for distinguishing ccRCC vs. not-ccRCC that the area under the receiver operating characteristic curve (AUC) of the ALL-P and CMP models was 0.89, but the overall sensitivity/specificity/accuracy of the ALL-P model was better. For cRCC vs. non-cRCC, the ALL-P model had the best performance.

**Conclusions:**

A reliable prediction model for RCC subtypes was constructed. The performance of the ALL-P prediction model was the best as compared to individual single-phase models and the traditional prediction model.

## 1. Introduction

Renal cell carcinoma (RCC) is one of the 10 most common malignant tumors in humans and the second most common malignant tumor of the urinary tract, accounting for about 3% of total cancers and 85% of malignant renal tumors [1]. Based on the 2016 World Health Organization (WHO) classification criteria, clear cell RCC (ccRCC), papillary RCC (pRCC), and chromophobe RCC (cRCC) account for 95% of all renal cancers and have a frequency of 75%, 15%, and 5%, respectively [2]. The biological behavior and aggressiveness of the different RCC subtypes are different and thus are treated differently and have a different prognosis [3]. Studies have shown that around 94% of patients with metastatic disease have ccRCC with a 5-year survival rate of 44% to 69%, while the 5-year survival rate of type I pRCC and cRCC ranges from 78% to 92% [4, 5]. Currently, less aggressive subtypes associated with a better prognosis are treated with a nephron-sparing partial nephrectomy and certain nonsurgical treatments [6].

The 2018 National Comprehensive Cancer Network (NCCN) guidelines for the treatment of renal cancer indicate that the first choice of treatment for patients with ccRCC who have stage IV disease, have had a relapse, or are not surgical candidates is axitinib plus pembrolizumab; the first choice of treatment for patients with non-ccRCC is sunitinib or participation in a clinical trial [7]. Targeted drugs and immunotherapy are also used to treat RCC, and the selection is based on the RCC subtype [8, 9]. As such, accurate identification of the RCC subtype is important for selecting the most appropriate treatment and improving outcomes.

Histopathological examination of a tissue specimen obtained by needle biopsy or surgical biopsy of the tumor nidus is the most accurate method for determining the RCC subtype; however, these methods are limited by the tumor location and their invasiveness. Noninvasive imaging methods such as computed tomography (CT) and functional magnetic resonance imaging (fMRI) have been studied with respect to diagnosis RCC and subtype, but study results have been inconsistent [10, 11].

Radiomic features (RFs) reflect the homogeneous phenomenon of the pixels in an image and can be quantitatively analyzed [12]. By combining medical imaging, gene analysis, and clinical data, RFs analysis can be used to extract and analyze tumor information in a high-throughput manner using artificial intelligence methods and provide a diagnosis that is much more accurate than the diagnosis that can be achieved with conventional imaging methods [13]. Although the RFs method has achieved some success for the prediction of the RCC subtype, certain issues remain to be solved. Most studies have used RFs obtained from 2-dimensional (2D) CT enhancement phases; however, RFs obtained by this method do not fully reflect tumor biological information as compared to data obtained by 3D CT. In addition, most studies have focused on 2-tier or single subtype prediction (i.e., benign or malignant, ccRCC or not-ccRCC, low or high grade) and thus are not useful for predicting the subtype in general. Lastly, most experiment datasets used in prior studies were balanced, and this does not reflect the actual distribution of the different subtypes.

The purpose of this study was to develop a reliable prediction model to distinguish RCC subtypes using 3D multiphase enhanced CT RFs using an unbalanced dataset reflecting the actual distribution of RCC subtypes.

## 2. Materials and Methods

### 2.1. Patients

This study used imaging data and clinical data retrospectively obtained from the medical records of patients with RCC treated at the Southern Medical University hospital from January 2013 to December 2018. This study was approved by the Ethics Committee of Southern Medical University, and because of the retrospective nature of the study, the requirement of informed patient consent was waived.

Inclusion criteria were as follows: (1) diagnosis of RCC confirmed by histopathological examination of a tissue specimen by 2 pathologists; (2) good CT image quality (clear image with no artifacts); (3) no treatment prior to the CT examination; and (4) CT performed with 4 phases: noncontrast phase (NCP), corticomedullary phase (CMP), nephrographic phase (NP), and excretory phase (EP). Exclusion criteria were as follows: (1) two or more lesions in a single kidney or lesion in both kidneys; (2) RCC with mixed features (e.g., papillary and clear cell features); and (3) RCC having most or all cysts features.

### 2.2. CT Acquisition

Two CT devices were used to examine patients during the study period: a 64-multidetector spiral CT scanner (Somatom definition CT, Siemens Medical Solutions) and a 256-multidetector spiral CT scanner (Brilliance ICT, Philips Medical Systems). In all patients, all CT images were obtained during breath-holding. Scans were performed with patients in the supine position, and the scanning range was from the phrenic top to the lower pole of both kidneys. CT parameters were as follows: tube voltage = 120 kV, tube current = 150–320 mA, layer thickness = 5 mm, interlayer spacing = 5 mm, field of view (FOV) = 360 mm, and matrix = 512 × 512. Spiral scanning and thin-slice reconstruction were performed for images obtained in each of the 4 phases. The CMP, NP, and EP CT scanning phases were begun at 30–35 s, 50–60 s, and 190–200 s, respectively, after the contrast agent was injected into the antecubital vein. The contrast agent used was either Omnipaque (GE Healthcare) or Ultravist (Bayer, Schering Pharma). The dosage was 2 ml/kg, and the agent was injected by a high-pressure injector at a rate of 2.5 ml/s, with a maximum volume of 160 ml. All images were examined on the picture archiving and communication system (PACS) at our hospital.

**Figure 1:**
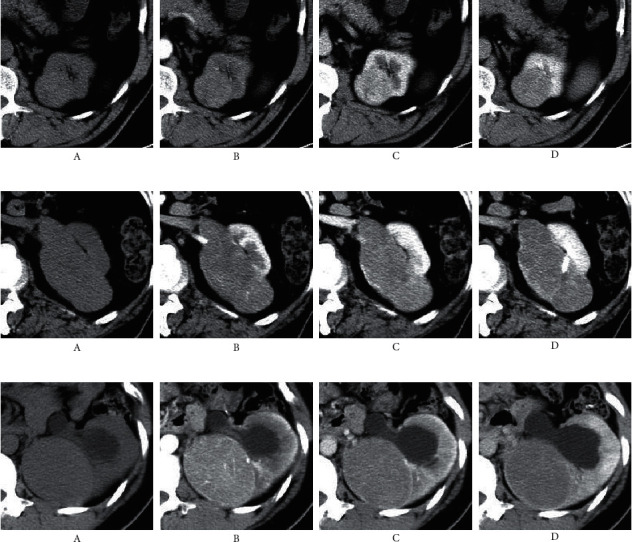
Enhanced computed tomography (CT) images of different subtypes of renal carcinoma in different phases. (a) Clear cell renal cell carcinoma; (b) papillary renal cell carcinoma; (c) chromophobe renal cell carcinoma. (A) Noncontrast phase; (B) corticomedullary phase; (C) nephrographic phase; (D) excretory phase.

Representative enhanced CT images of RCC at different phases are shown in Figure 1.

### 2.3. RFs Segmentation

**Figure 2:**
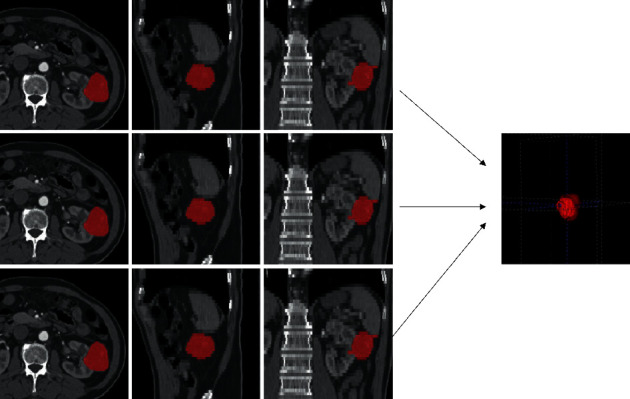
Computed tomography (CT) image segmentation in a corticomedullary phase enhanced CT scan.

All patient images extracted from the PACS were anonymized. A CT image from each phase with a layer thickness of 5 mm was chosen for analysis. CT images were required to have a window width of 300 to 400 HU and a window level of 45 to 65 HU. Each image was segmented with ITK-SNAP software (http://www.itk-snap.org) independently by 2 radiologists with 10 and 15 years of experience, respectively, in the diagnosis of abdominal imaging. Because NCP images do not clearly show lesion boundaries, segmentation of the CMP was used to represent that of the NCP image. A 3D volume of tumor tissue was selected as the region of interest (ROI). The ROI was manually drawn along the irregular contour of the tumor and was kept approximately 2 mm from the margin of the tumor to reduce interference by adjacent tissues, such as fat or normal renal tissue [14]. An example of image segmentation is shown in Figure 2.

### 2.4. RFs Extraction

The PyRadiomics computing platform [13], which uses a large panel of engineered hard-coded features algorithms, was used for the extraction and processing of RFs from medical images. In brief, a segmented image was loaded and preprocessed, bad RFs were filtered, and finally, the numbers of RFs were calculated and grouped into different feature classes. To ensure the stability and reproducibility of the results, the interclass correlation coefficient (ICC) was calculated with respect to the RFs examined by the 2 radiologists. RFs with an ICC > 0.80 were regarded as being in good agreement and retained for further analysis.

### 2.5. RF Selection and Model Construction

**Figure 3:**
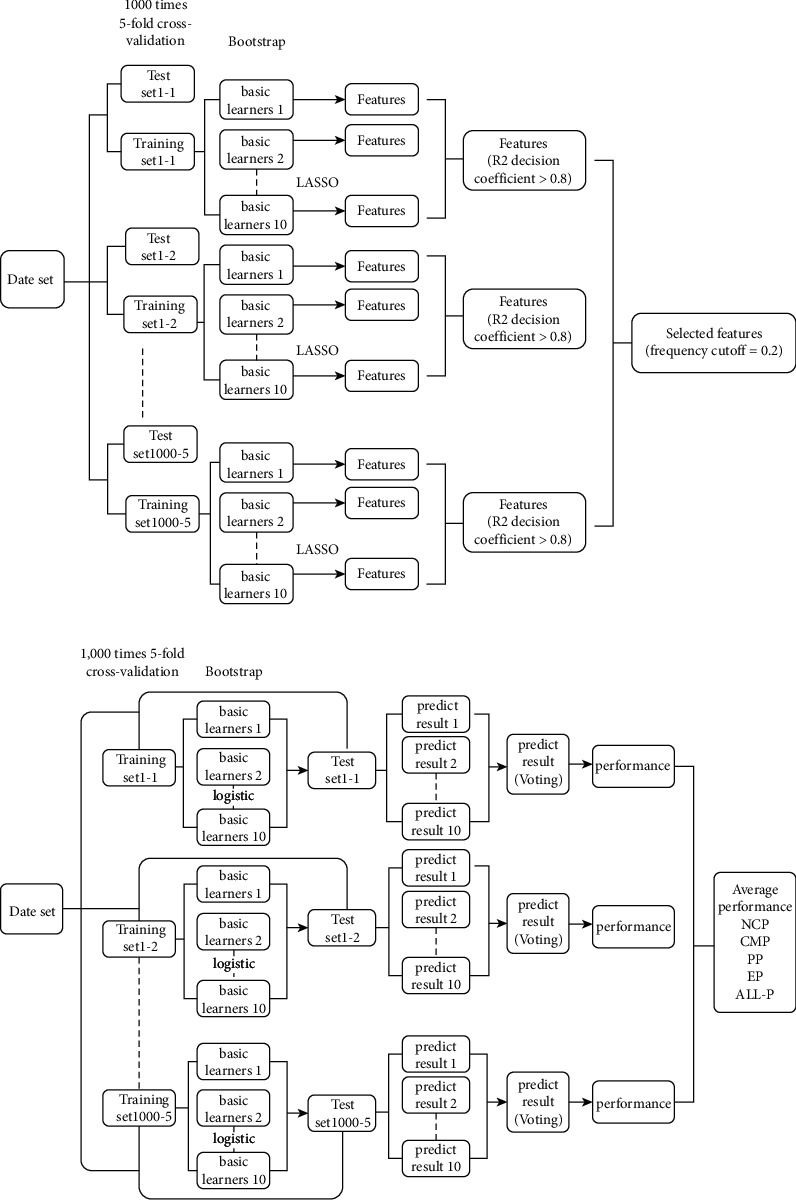
(a) Flow chart of the feature selection based on the ensemble learning bagging method. (b) Flow chart of the model construction based on the ensemble learning bagging method.

The ensemble learning bagging method was used to classify the different RCC subtypes. The LASSO regression method was chosen as the feature selection model basic learner [15]. Ten basic learners were set, and each basic learner processed 1,000, 5-fold cross-validations. The 5-fold cross-validation method combines bootstrap and traditional validation (training set vs. validation set). In each trial/model, the whole dataset is randomly divided into a training set (80% of samples) and a validation set (20% of samples). All samples are then in turn allocated into the training set and validation set in one complete 5-fold cross-validation. The bootstrap method reduces the influence of a small sample size by performing a large number of simulations, which achieves a consistent estimation. All trained models with an *R*^2^ decision coefficient >0.8 were retained. The distribution of imaging features was analyzed, and the most significant imaging features (frequency cutoff = 0.2) were selected (Figure 3(a)). The selected features were used as the input features in their own final classification model.

The One-vs.-Rest (OvR: also referred to as One-vs.-All or OvA) method was used as the basic learner to refit the final prediction models of single-phase and all-phase studies (NCP/CMP/NP/EP/ALL-P). Fivefold and external stratified cross-validation was used to assess the performance of the classification models with the quantitative indices of the area under the receiver operating characteristic curve (AUC), sensitivity, specificity, positive predictive value, negative predictive value, accuracy, precision, and *f*1 score. The performance of the models (AUC) was expressed as a mean value of 1000 times 5-fold-cross-validation.

A flow chart of the subtype prediction model is shown in Figure 3(b). This method was used to construct a 3-category classification prediction model for the ccRCC, pRCC, and cRCC subtypes. In order to more intuitively illustrate the discrimination of each subtype using the RFs model, binary classification prediction of the different RCC subtypes was also performed, i.e., ccRCC/not-ccRCC, pRCC/not-pRCC, and cRCC/not-cRCC.

### 2.6. Traditional Prediction Method

A radiologist with 10 years of experience in the interpretation of abdominal imaging evaluated each case based on traditional imaging studies and clinical data. Data extracted from the medical records and imaging studies included patient age, sex, and clinical symptoms, tumor side (left or right), tumor location (inside: the tumor was completely within the contour of the kidney, middle: the tumor was more than 50% inside the contour of the kidney, outside: the tumor was more than 50% outside the contour of the kidney), the presence of cystic components and calcifications, and TNM stage. The maximum diameter of the tumor and the CT attenuation of the solid portion of the tumor at the maximum diameter were measured. The 5-fold cross-validation method was used to establish a logistic regression equation to predict the RCC subtype by selecting variables with a *p* < 0.05. The logistic regression model composed of the aforementioned data served as a control model. The performance of the model is expressed as an average value.

### 2.7. Statistical Analysis

Statistical analyses were conducted using SPSS version 22.0 software. The nonparametric Kruskal-Wallis *H* test was used for the comparison measurement data that did not conform to a normal distribution, and the *χ*^2^ test was used for the examination of classification variables (those with a value of *p* < 0.05). Python was used to draw the ROC curves and prepare the confusion matrix (i.e., a display of model prediction results) of the models. Comparisons of ROC curves and AUC of multiple models were performed using the Delong test of MedCalc software.

## 3. Results

### 3.1. Patients

Data of 261 patients with RCC were included in the analysis, and characteristics are summarized in Table 1. Of them, 162 cases were scanned by a 64-multidetector spiral CT scanner while 99 cases were scanned by a 256-multidetector spiral CT scanner. Patient groups with the 3 different RCC subtypes were not significantly different with respect to age (*p*=0.786), tumor side (*p*=0.946), tumor diameter (*p*=0.708), symptoms (*p*=0.385), tumor growth (*p*=0.879), tumor calcifications (*p*=0.195), T stage (*p*=0.748), M stage (*p*=0.073), TNM stage (*p*=0.195), and NCP lesion attenuation (*p*=0.185).

### 3.2. RF Extraction and Selection

A total of 105 RFs were extracted from the 3D multiphase enhanced CT images of each phase of each patient. The RF features were categorized as follows: (1) first-order statistics, (2) shape-based (3D) features, and (3) texture features. Of the 105 RFs, there were 18 first-order statistics features, 13 shape-based (3D) features, and 74 texture features (23 gray level cooccurrence matrix (GLCM) features, 16 gray level size zone matrix (GLSZM) features, 16 gray level run length matrix (GLRLM) features, 14 gray level dependence matrix (GLDM) features, and 5 neighboring gray-tone difference matrix (NGTDM) features). Based on the ensemble learning stratified bagging method and the LASSO regression algorithm, RFs that were significant in each of the 4 single-phase (NCP, CMP, NP, and EP) images were used to create a model for each of the 4 phases, respectively. In addition, an all-phase model (ALL-P) was produced by reextracting the features of all 4 phases together.

The RFs of every model and *p* values are shown in Supplemental Table 1.

### 3.3. Prediction Performance

The average performance of the RF models and the traditional prediction model for predicting the RCC subtype is shown in Tables 2 and 3. The ALL-P model performed the best of all models, with an average accuracy of 0.80. Using the ALL-P model, the average sensitivity and specificity for ccRCC were 0.85 and 0.83; those for pRCC were 0.60 and 0.91; those for cRCC were 0.66 and 0.91, respectively.

The traditional prediction model used data of sex, lesion cystic features, N stage, and lesion attenuation in the CMP, NP, and EP phases, and variables with a value of *p* < 0.05 were included in the logistic regression model. The average accuracy of the traditional prediction model was 0.81; the average sensitivity and specificity for ccRCC were 0.97 and 0.30; those for pRCC were 0.21 and 0.97; those for cRCC were 0.12 and 0.97, respectively.

**Figure 4:**
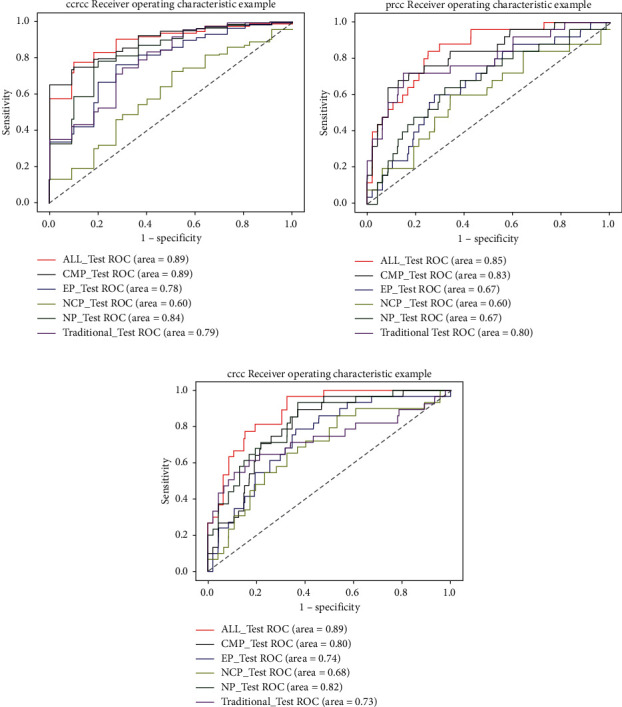
Receiver operating characteristic (ROC) curves of the 3 subtype prediction models and traditional prediction model from the single subtype binary classification experiments. (a) ROC curves of ccRCC prediction models and the traditional model in the single subtype binary classification experiments. (b) ROC curves of cRCC prediction models and the traditional model in the single subtype binary classification experiments. (c) ROC curves of pRCC prediction models and the traditional model in the single subtype binary classification experiments. cRCC, chromophobe cell renal cell carcinoma; ccRCC, clear cell renal cell carcinoma; pRCC, papillary cell renal cell carcinoma.

The results of single RCC subtype binary classification (ccRCC/not-ccRCC; pRCC/not-pRCC; cRCC/not-ccRCC) using RF models and the traditional model are shown in Table 4 and Figure 4. In the classification of ccRCC vs. not-ccRCC, the average performance of the ALL-P and CMP models was the best (both, AUC = 0.89) and better than the average performance of the traditional prediction model (AUC = 0.79). The performance of the ALL-P and CMP models was not statistically different (*p*=0.98), and the performance of both models was significantly better than that of the traditional model (both, *p* < 0.001).

In the classification of pRCC vs. not-pRCC, the average performance of the ALL-P was the best (AUC = 0.85), followed by the CMP model (AUC = 0.83), and then the traditional prediction model (AUC = 0.80). However, the performance of the 3 models was not significantly different (*p*=0.09,  *p*=0.13). The overall average accuracy of the traditional model was the highest. In the classification of cRCC vs. not-cRCC, the average performance of the ALL-P model was the best (AUC = 0.89), and this was significantly better than that of the NP model (AUC = 0.82) and the traditional prediction model (AUC = 0.73) (*p*=0.03,  *p* < 0.001, respectively).

## 4. Discussion

Treatment of RCC is challenging because RCC is genetically diverse, and the different subtypes have different prognoses and respond differently to treatments. Even after staging and grading control, ccRCC has a worse prognosis than pRCC and cRCC [16, 17]. It has been reported that 5-year tumor-specific survival rates (CSS) for TNM stage I, II, III, and IV ccRCC patients (1987–1998) were 91%, 74%, 67%, and 32%, respectively [18]. The pRCC has a good prognosis and more than 75% of cases can be treated by nephron-sparing surgery [19]. The cRCC usually has the best prognosis, with a 5-year relapse-free survival of 89.3% and a 10-year CSS of 88.9% [20]. Thus, to accurately treat RCC and optimize outcomes, it is necessary to correctly determine the RCC subtype [21].

RFs analysis is widely used in the diagnosis of lung, breast, liver, and colorectal cancers, but relatively few studies have explored its use with respect to RCC [22]. In this study, we use 3D RFs which can represent tumor heterogeneity much better than 2D RFs because the whole tumor is analyzed, rather than only a single slice. Prior study has indicated that whole tumor analysis provides a better representation of tumor heterogeneity than when representative slices are examined [23]. In this study, we developed models to distinguish between the 3 most common RCC subtypes based on RFs and found that the ALL-P RF model, which contains complete information regarding tumor blood flow and dynamically reflects tumor heterogeneity, provided the best predictive value and was markedly better than that of the traditional prediction model. Importantly, most prior studies of RCC have focused on distinguishing RCC subtypes from benign tumors and were only based on single-phase RFs. Commonly used machine learning models include Decision Tree, Random Forest, Support Vector Machine (SVM), Artificial Neural Networks, Clustering Analysis, Deep Learning, and Bayesian Learning. The logistic regression model is the most common classification model due to its high stability. The SVM models were the most common method to differentiate renal tumors using CT radiomics [24, 25]. In previous studies [26, 27], sample data were divided into a training set and test set once time, which would lead to result errors due to sampling division. Kocak et al. [28] have reported that CMP CT images provide much more valuable texture parameters than NCP images to predict RCC subtypes. Hodgdon et al. [29] reported that CT texture analysis of NP can be used to distinguish fat-poor angiomyolipoma (fp-AML) and ccRCC. Coy et al. [30] have reported that the best classification result for ccRCC and oncocytoma on multiphase CT was obtained in EP. Zhou et al. have [31] reported that the radiomic features from the unenhanced, corticomedullary phase, and nephrographic phase can effectively distinguish the four nuclear grades. A combination of the Random Forest model, merging radiomic features, and clinical characteristics achieved good predictive performance in the internal test set and external test set.

In this study, the LASSO regression method was used to analyze the distributions of RFs in different phases in order to determine which RFs were most important. This is somewhat different from the traditional method because when the lasso is used to select the features, many will have *p* values >0.05. However, a *p* value >0.05 only means “no evidence of difference;” it does not mean “evidence of no difference.” As such, the inclusion of RFs should not be based solely on the *p* value, and this study illustrates that the ensemble learning bagging method may identify useful information that is not identified using traditional statistical methods, and that using a lasso to select features provides good results.

In this study, the dataset that was used was small and unbalanced, and the distribution of RCC subtypes was close to the actual distribution reported in the literature. To avoid issues involved in the analysis of small and/or unbalanced datasets, the ensemble learning bagging method was used. The ensemble learning bagging method is a machine learning paradigm where multiple models (often called “basic learners” or “weak learners”) are trained to solve the same problem, and then, the models are combined to provide results with increased accuracy [32]. The bagging method uses the bootstrap method [33] to make certain that the selection of all the samples in the dataset has the same probability and the same distribution. Using the LASSO regression algorithm as the base learner simplifies the complexity of a prediction model and can determine which features are most important in the prediction model. Subsequently, the One-vs.-Rest logistic regression algorithm method and external layered cross-validation were used to refit the prediction models, which ensured the reliability of the prediction result.

Results of the comparison of the predictive performance of the 5 RFs models and the traditional prediction model indicated that the ALL-P performed the best with respect to distinguishing between the 3 RCC subtypes. The ALL-P model exhibited the highest sensitivity and specificity for each RCC subtype. Although the AUC of the traditional model was slightly higher than that of the ALL-P model (0.81 vs. 0.80), the traditional model exhibited very low sensitivity for predicting cRCC and pRCC and very low specificity for predicting ccRCC. As such, the traditional model offered little value for the prediction of pRCC and cRCC subtype, which is due to the unbalanced subtype distribution.

Binary classification experiments showed for distinguishing ccRCC vs. not-ccRCC that the area under the receiver operating characteristic curve (AUC) of the ALL-P and CMP models was 0.89, but the overall sensitivity/specificity/accuracy of the ALL-P model was better; for pRCC vs. not-pRCC, the AUC of the ALL-P was the highest (AUC = 0.85). However, there was no significant difference with the traditional prediction model (AUC = 0.80) (*p*=0.13); the overall accuracy of the traditional model was the highest; for cRCC vs. non-cRCC, the ALL-P model offered statistically better performance than the other models.

There are several limitations of this study that should be considered. First, the dataset was relatively small; however, methods of analysis were used to account for this. In future research, we plan to study this topic with a larger dataset. Second, only a linear basic learner was used; the use of different learners which may better suit RCC subtype data may improve the prediction performance. Third, this study used a retrospectively obtained dataset; validation of the method in a prospective study is necessary to determine the true predictive performance of the method.

## 5. Conclusions

In conclusion, we constructed a reliable prediction model based on 3D multiphase enhanced CT image features to predict the 3 most common RCC subtypes. The performance of the ALL-P prediction model was the best as compared to individual single-phase models and the traditional prediction model. This noninvasive prediction model may help guide treatment decisions and precise treatment.

## Figures and Tables

**Table 1:** Characteristics of patients and renal lesions.

	RCC			*p*
	ccRCC (*n* = 209)	pRCC (*n* = 25)	cRCC (*n* = 29)	
Sex				0.024
Male	136 (65.6%)	18 (72%)	12 (41.4%)	
Female	71 (34.4%)	7 (28%)	17 (58.6%)	
Age	52	52	54	0.786
Side		0.946		
Right	114 (55%)	10 (40%)	13 (44.8%)	
Left	93 (45%)	15 (60%)	16 (55.2%)	
Diameter	45.07	45.23	47.36	0.708
Symptoms				0.385
−	91 (44%)	10 (40%)	17 (58.6)	
+	116 (56%)	15 (60%)	12 (41.4)	
Growth				0.879
Outside	70 (33.5%)	7 (28%)	10 (34.5%)	
Middle	90 (43.5%)	10 (40%)	13 (44.8%)	
Inside	47 (23.0%)	8 (32%)	6 (20.7%)	
Cystic				0.031
−	46 (22.5%)	14 (56)	20 (69%)	
+	161 (91.6%)	11 (44%)	9 (31%)	
Calcification				0.195
−	162 (78.2%)	21 (84%)	27 (93.1%)	
+	42 (22.8%)	4 (16%)	2 (6.9%)	
T stage				0.748
T1	144 (69.5%)	15 (60%)	21 (72.4%)	
T2	25 (12.1%)	5 (20%)	6 (20.7%)	
T3	17 (8.2%)	2 (8%)	1 (3.4%)	
T4	21 (10.2%)	3 (12%)	1 (3.4%)	
N stage				<0.001
N0	186 (89.8%)	19 (76%)	28 (96.6%)	
N1	22 (10.2%)	6 (24%)	1 (3.4%)	
M stage				0.073
M0	194 (93.7%)	23 (92%)	27 (93.1%)	
M1	13 (6.2%)	2 (8%)	2 (6.9%)	
TNM				0.195
I	130 (62.8%)	13 (52%)	20 (69%)	
II	26 (12.6%)	2 (8%)	6 (20.7%)	
III	13 (6.3%)	6 (24%)	2 (6.9%)	
IV	28 (18.3%)	4 (16%)	1 (3.4%)	
Lesion attenuation				
NCP	33.89	35.35	26.61	0.185
CMP	105.31	53.61	85.21	<0.001
NP	86.98	61.9	77.81	<0.001
EP	69.93	60.71	65.95	<0.001

CMP, corticomedullary phase; EP, excretory phase; NCP, noncontrast phase; NP, nephrographic phase. Data are presented as numbers (percentage) or median. Lesion attenuation is reported as Hounsfield units. *p* values were calculated by *T* test, Kruskal-Wallis test, and *χ*^2^ test.

**Table 2:** Confusion matrix of the radiomic feature prediction models and traditional prediction method for the classification of the 3 RCC subtypes.

Test set		ccRCC	Pathology pRCC	cRCC
NCP	ccRCC	129	13	9
	pRCC	38	6	3
	cRCC	40	6	17

CMP	ccRCC	165	4	3
	pRCC	17	10	9
	cRCC	25	11	17

NP	ccRCC	159	11	10
	pRCC	34	4	6
	cRCC	14	10	13

EP	ccRCC	146	9	8
	pRCC	31	8	10
	cRCC	30	8	11

ALL-P	ccRCC	175	5	4
	pRCC	16	15	6
	cRCC	16	5	19

Traditional	ccRCC	201	18	20
	pRCC	3	3	3
	cRCC	3	4	6

ALL-P, all-phase; CMP, corticomedullary phase; cRCC, chromophobe cell renal cell carcinoma; ccRCC, clear cell renal cell carcinoma; EP, excretory phase; NCP, noncontrast phase; NP, nephrographic phase; pRCC, papillary cell renal cell carcinoma. Data indicate the number of lesions.

**Table 3:** Average performance of the radiomic feature prediction models and traditional prediction method for the classification of 3 RCC subtypes in the test set.

		Sensitivity	Specificity	Precision	*f*1 score	Accuracy
NCP	ccRCC	0.62	0.59	0.85	0.72	0.58
	pRCC	0.24	0.83	0.13	0.17	
	cRCC	0.59	0.80	0.27	0.37	

CMP	ccRCC	0.80	0.89	0.96	0.87	0.73
	pRCC	0.40	0.89	0.28	0.41	
	cRCC	0.59	0.93	0.32	0.33	

NP	ccRCC	0.77	0.61	0.88	0.82	0.67
	pRCC	0.16	0.83	0.09	0.39	
	cRCC	0.45	0.89	0.35	0.12	

EP	ccRCC	0.7	0.69	0.90	0.79	0.63
	pRCC	0.32	0.83	0.16	0.22	
	cRCC	0.38	0.84	0.22	0.28	

ALL-P	ccRCC	0.85	0.83	0.95	0.88	0.80
	pRCC	0.60	0.91	0.41	0.49	
	cRCC	0.66	0.91	0.48	0.56	

Traditional	ccRCC	0.97	0.30	0.84	0.90	0.81
	pRCC	0.21	0.97	0.46	0.29	
	cRCC	0.12	0.97	0.33	0.18	

ALL-P, all-phase; CMP, corticomedullary phase; cRCC, chromophobe cell renal cell carcinoma; ccRCC, clear cell renal cell carcinoma; EP, excretory phase; NCP, noncontrast phase; NP, nephrographic phase; pRCC, papillary cell renal cell carcinoma.

**Table 4:** Average performance of the 5 prediction models and the traditional model for single subtype binary classification in the test set.

		Sensitivity	Specificity	Positive predictive value	Negative predictive value	Accuracy	AUC	*p*
ccRCC vs. not-ccRCC	NCP	0.63	0.52	0.83	0.27	0.61	0.60	<0.001
	CMP	0.82	0.80	0.94	0.53	0.81	0.89	0.98
	NP	0.77	0.72	0.91	0.45	0.76	0.84	0.02
	EP	0.75	0.65	0.89	0.4	0.73	0.78	<0.001
	ALL-P	0.83	0.85	0.96	0.57	0.84	0.89	
	Traditional	0.86	0.61	0.85	0.53	0.80	0.79	<0.001

pRCC vs. not-pRCC	NCP	0.48	0.73	0.16	0.93	0.70	0.60	<0.001
	CMP	0.68	0.76	0.23	0.96	0.75	0.83	0.09
	NP	0.4	0.75	0.14	0.92	0.72	0.67	<0.001
	EP	0.48	0.73	0.16	0.93	0.70	0.67	<0.001
	ALL-P	0.76	0.81	0.29	0.97	0.8	0.85	
	Traditional	0.84	0.67	0.17	0.91	0.89	0.80	0.13

cRCC vs. not-ccRCC	NCP	0.59	0.70	0.20	0.93	0.69	0.68	<0.001
	CMP	0.83	0.74	0.29	0.97	0.75	0.80	<0.001
	NP	0.76	0.78	0.31	0.96	0.78	0.82	0.03
	EP	0.62	0.74	0.23	0.94	0.73	0.74	<0.001
	ALL-P	0.93	0.84	0.42	0.99	0.85	0.89	
	Traditional	0.59	0.76	0.38	0.90	0.88	0.73	<0.001

ALL-P, all-phase; AUC, area under the receiver operating characteristic (ROC) curve; CMP, corticomedullary phase; cRCC, chromophobe cell renal cell carcinoma; ccRCC, clear cell renal cell carcinoma; EP, excretory phase; NCP, noncontrast phase; NP, nephrographic phase; pRCC, papillary cell renal cell carcinoma. All *p* values are unadjusted and were calculated in comparison with the ALL-P model.

## Data Availability

All the data and materials are included within the main paper.

## Supplementary Materials

Supplementary Materials Supplemental Table 1: radiomic features of the single-phase models and all-phase model.
