# Supplementary material for: A Reliable Prediction Model for Renal Cell Carcinoma Subtype Based on Radiomic Features from 3D Multiphase Enhanced CT Images
Source: J Oncol. 2021 Sep 21;2021:6595212. doi: 10.1155/2021/6595212 (PMC8478553; doi:10.1155/2021/6595212)
Supplement: Supplementary Materials — Supplemental Table 1: radiomic features of the single-phase models and all-phase model. [file 6595212.f1.docx]

**Supplemental Table 1. Radiomics features of the single-phase models and all-phase model**

| **Phase of CT scan** | **ccRCC** | ***P*** | **pRCC** | ***P*** | **cRCC** | ***P*** |
| --- | --- | --- | --- | --- | --- | --- |
| **NCP** | firstorder_10Percentile | 0.011 | firstorder_InterquartileRange | 0.191 | firstorder_MeanAbsoluteDeviation | 0.913 |
|  | firstorder_Minimum | 0.785 | firstorder_Median | 0.224 | firstorder_Median | 0.001 |
|  | glcm_Idmn | 0.603 | glcm_Imc1 | 0.053 | firstorder_RootMeanSquared | <0.001 |
|  | glcm_Idmn | 0.032 | glcm_MaximumProbability | 0.101 | glcm_ClusterTendency | 0.954 |
|  | glcm_InverseVariance | 0.001 | gldm_DependenceVariance | 0.796 | glcm_Idmn | 0.493 |
|  | glcm_MCC | 0.081 | gldm_LargeDependenceHighGrayLevelEmphasis | 0.236 | glcm_Imc1 | 0.493 |
|  | gldm_DependenceEntropy | <0.001 | glrlm_GrayLevelNonUniformityNormalized | 0.408 | glcm_MaximumProbability | 0.002 |
|  | glrlm_GrayLevelNonUniformityNormalized | 0.833 | glrlm_LongRunLowGrayLevelEmphasis | 0.355 | gldm_DependenceVariance | 0.001 |
|  | glrlm_RunVariance | 0.061 | glrlm_RunLengthNonUniformity | 0.620 | glrlm_RunEntropy | 0.048 |
|  | glszm_GrayLevelNonUniformityNormalized | 0.366 | glszm_LargeAreaEmphasis | 0.998 | glrlm_ShortRunLowGrayLevelEmphasis | 0.869 |
|  | shape_Sphericity | 0.544 | glszm_LowGrayLevelZoneEmphasis | 0.079 | glszm_ZonePercentage | 0.010 |
|  |  |  | glszm_SmallAreaLowGrayLevelEmphasis | 0.084 | ngtdm_Coarseness | 0.213 |
|  |  |  | glszm_ZoneEntropy | 0.325 | shape_Sphericity | 0.336 |
|  |  |  | ngtdm_Contrast | 0.976 | shape_SurfaceVolumeRatio | 0.376 |
|  |  |  |  |  |  |  |
| **CMP** | firstorder_Skewness | 0.008 | glszm_ZoneEntropy | <0.001 | firstorder_Median | 0.550 |
|  | glcm_Imc1 | <0.001 | firstorder_Minimum | 0.071 | firstorder_Minimum | 0.204 |
|  | glcm_InverseVariance | <0.001 | glcm_Id | <0.001 | glcm_Idm | <0.001 |
|  | glcm_JointEntropy | <0.001 | glcm_Idm | <0.001 | glcm_Idmn | 0.089 |
|  | gldm_DependenceEntropy | <0.001 | gldm_DependenceVariance | <0.001 | glcm_Imc1 | 0.001 |
|  | gldm_DependenceNonUniformityNormalized | <0.001 | gldm_SmallDependenceEmphasis | 0.021 | glcm_InverseVariance | 0.074 |
|  | glrlm_LongRunEmphasis | <0.001 | glszm_SmallAreaEmphasis | 0.180 | gldm_DependenceEntropy | <0.001 |
|  | glrlm_RunPercentage | <0.001 | ngtdm_Coarseness | 0.878 | glrlm_GrayLevelNonUniformityNormalized | <0.001 |
|  | glszm_GrayLevelNonUniformityNormalized | <0.001 | ngtdm_Contrast | <0.001 | glrlm_RunEntropy | 0.002 |
|  | glszm_LargeAreaLowGrayLevelEmphasis | 0.071 | shape_SurfaceVolumeRatio | 0.815 | glrlm_RunPercentage | <0.001 |
|  | shape_Sphericity | 0.293 |  |  | glszm_SmallAreaEmphasis | 0.756 |
|  |  |  |  |  | glszm_ZoneEntropy | 0.020 |
|  |  |  |  |  | glszm_ZonePercentage | 0.420 |
|  |  |  |  |  | ngtdm_Contrast | <0.001 |
|  |  |  |  |  | shape_Sphericity | 0.587 |
|  |  |  |  |  | shape_SurfaceVolumeRatio | 0.365 |
|  |  |  |  |  |  |  |
| **NP** | firstorder_10Percentile | 0.907 | firstorder_Skewness | 0.732 | firstorder_90Percentile | 0.076 |
|  | firstorder_Median | 0.001 | glcm_DifferenceVariance | 0.016 | firstorder_MeanAbsoluteDeviation | 0.001 |
|  | glcm_Correlation | <0.001 | glcm_Imc1 | 0.167 | firstorder_Minimum | 0.282 |
|  | glcm_Idmn | 0.335 | glcm_JointEnergy | <0.001 | firstorder_TotalEnergy | 0.266 |
|  | glcm_Imc1 | 0.006 | glcm_MaximumProbability | <0.001 | firstorder_Variance | 0.008 |
|  | glcm_InverseVariance | <0.001 | gldm_DependenceVariance | 0.049 | glcm_DifferenceAverage | 0.005 |
|  | glcm_JointEntropy | <0.001 | glrlm_GrayLevelNonUniformityNormalized | 0.004 | glcm_JointEnergy | <0.001 |
|  | gldm_DependenceEntropy | <0.001 | glrlm_LongRunEmphasis | 0.079 | glcm_MaximumProbability | <0.001 |
|  | gldm_DependenceNonUniformityNormalized | <0.001 | glszm_GrayLevelNonUniformity | 0.757 | gldm_DependenceNonUniformityNormalized | <0.001 |
|  | glrlm_LongRunHighGrayLevelEmphasis | 0.207 | glszm_GrayLevelNonUniformityNormalized | 0.016 | gldm_DependenceVariance | <0.001 |
|  | glszm_GrayLevelNonUniformityNormalized | <0.001 | glszm_SmallAreaLowGrayLevelEmphasis | 0.648 | gldm_GrayLevelVariance | 0.007 |
|  | glszm_LargeAreaLowGrayLevelEmphasis | 0.319 | glszm_ZoneEntropy | 0.087 | glrlm_GrayLevelNonUniformityNormalized | <0.001 |
|  | glszm_LowGrayLevelZoneEmphasis | 0.396 | glszm_ZonePercentage | 0.918 | shape_Flatness | 0.734 |
|  | glszm_SmallAreaEmphasis | 0.587 | ngtdm_Coarseness | 0.748 | shape_SurfaceVolumeRatio | 0.281 |
|  | shape_Sphericity | 0.459 | shape_SurfaceArea | 0.749 |  |  |
|  |  |  | shape_SurfaceVolumeRatio | 0.817 |  |  |
|  |  |  |  |  |  |  |
| **EP** | firstorder_10Percentile | 0.661 | firstorder_Kurtosis | 0.300 | firstorder_InterquartileRange | <0.001 |
|  | firstorder_Median | 0.041 | glcm_JointAverage | 0.638 | firstorder_Kurtosis | 0.035 |
|  | firstorder_Minimum | 0.159 | glcm_JointEnergy | 0.004 | firstorder_MeanAbsoluteDeviation | 0.021 |
|  | glcm_Correlation | 0.001 | glcm_MCC | 0.909 | glcm_Imc1 | 0.075 |
|  | glcm_Idmn | 0.929 | glcm_SumAverage | 0.638 | glcm_JointAverage | 0.580 |
|  | glcm_InverseVariance | <0.001 | gldm_LargeDependenceLowGrayLevelEmphasis | 0.694 | glcm_JointEnergy | <0.001 |
|  | gldm_DependenceEntropy | <0.001 | glrlm_GrayLevelNonUniformityNormalized | 0.011 | glcm_MaximumProbability | 0.002 |
|  | glrlm_LongRunHighGrayLevelEmphasis | 0.090 | glrlm_LongRunLowGrayLevelEmphasis | 0.345 | glcm_SumAverage | 0.580 |
|  | glszm_LargeAreaEmphasis | 0.918 | glrlm_RunLengthNonUniformityNormalized | 0.125 | glrlm_LongRunLowGrayLevelEmphasis | 0.300 |
|  | glszm_LargeAreaLowGrayLevelEmphasis | 0.886 | glrlm_RunVariance | 0.065 | glrlm_RunLengthNonUniformityNormalized | 0.271 |
|  | ngtdm_Coarseness | 0.578 | glszm_LowGrayLevelZoneEmphasis | 0.984 | glrlm_RunVariance | 0.071 |
|  | shape_Elongation | 0.064 | glszm_SizeZoneNonUniformityNormalized | 0.329 | glszm_GrayLevelNonUniformityNormalized | 0.029 |
|  | shape_Flatness | 0.050 | shape_SurfaceArea | 0.798 | ngtdm_Contrast | 0.462 |
|  | shape_Maximum2DDiameterSlice | 0.279 |  |  |  |  |
|  | shape_Sphericity | 0.055 |  |  |  |  |
|  |  |  |  |  |  |  |
| **ALL-P** | CMP_glcm_JointEntropy | <0.001 | CMP_firstorder_Kurtosis | <0.001 | CMP_glcm_Idm | <0.001 |
|  | CMP_glszm_GrayLevelNonUniformityNormalized | <0.001 | CMP_firstorder_Uniformity | <0.001 | CMP_glcm_InverseVariance | 0.074 |
|  | CMP_glszm_SmallAreaLowGrayLevelEmphasis | 0.377 | CMP_glcm_Idn | 0.002 | CMP_glcm_MaximumProbability | <0.001 |
|  | EP_firstorder_Minimum | 0.159 | CMP_glcm_JointEnergy | <0.001 | CMP_gldm_DependenceVariance | <0.001 |
|  | EP_glcm_Idmn | 0.929 | CMP_glcm_MaximumProbability | <0.001 | CMP_glszm_ZoneEntropy | 0.020 |
|  | EP_glcm_InverseVariance | <0.001 | CMP_glszm_GrayLevelNonUniformity | 0.913 | EP_glcm_JointAverage | 0.580 |
|  | EP_gldm_DependenceEntropy | <0.001 | NCP_firstorder_InterquartileRange | 0.191 | EP_glrlm_GrayLevelNonUniformityNormalized | 0.031 |
|  | EP_glrlm_RunLengthNonUniformityNormalized | 0.049 | NCP_glrlm_ShortRunLowGrayLevelEmphasis | 0.071 | NCP_glcm_DifferenceEntropy | 0.528 |
|  | EP_shape_Sphericity | 0.055 | NCP_ngtdm_Busyness | 0.783 | NCP_gldm_DependenceVariance | 0.001 |
|  | NCP_glcm_Idmn | 0.603 | NP_gldm_SmallDependenceLowGrayLevelEmphasis | 0.878 | NCP_glszm_ZonePercentage | 0.010 |
|  | NCP_glszm_GrayLevelNonUniformityNormalized | 0.366 | NP_ngtdm_Coarseness | 0.748 | NCP_shape_Sphericity | 0.336 |
|  | NP_glcm_Imc1 | 0.006 |  |  | NP_firstorder_90Percentile | 0.076 |
|  | NP_glcm_InverseVariance | <0.001 |  |  | NP_firstorder_InterquartileRange | <0.001 |
|  | NP_gldm_DependenceEntropy | <0.001 |  |  | NP_glcm_JointEnergy | <0.001 |
|  |  |  |  |  | NP_glcm_SumAverage | 0.214 |
|  |  |  |  |  | NP_gldm_DependenceVariance | <0.001 |

ALL-P, all-phase; CMP, cortico-medullary phase; cRCC, chromophobe cell renal cell carcinoma; ccRCC, clear cell renal cell carcinoma; EP, excretory phase; NCP, non-contrast phase; NP, nephrographic phase; pRCC, papillary cell renal cell carcinoma.
